# Indole Alkaloids from the Leaves of *Nauclea officinalis*

**DOI:** 10.3390/molecules21080968

**Published:** 2016-07-23

**Authors:** Long Fan, Cheng-Hui Liao, Qiang-Rong Kang, Kai Zheng, Ying-Chun Jiang, Zhen-Dan He

**Affiliations:** Department of Pharmacy, School of Medicine, Shenzhen University, Shenzhen 518060, China; fanlong_kkk@163.com (L.F.); liaochenghui1990@163.com (C.-H.L.); kangqiangrong@163.com (Q.-R.K.); y.k.zheng@163.com (K.Z.); Yingchunjiang313@gmail.com (Y.-C.J.)

**Keywords:** indole alkaloids, *Nauclea officinalis*, biosynthetic pathway

## Abstract

Three new indole alkaloids, named naucleamide G (**1**), and nauclealomide B and C (**5** and **6**), were isolated from the *n*-BuOH-soluble fraction of an EtOH extract of the leaves of *Nauclea officinalis*, together with three known alkaloids, paratunamide C (**2**), paratunamide D (**3**) and paratunamide A (**4**). The structures with absolute configurations of the new compounds were identified on the basis of 1D and 2D NMR, HRESIMS, acid hydrolysis and quantum chemical circular dichroism (CD) calculation. According to the structures of isolated indole alkaloids, their plausible biosynthetic pathway was deduced.

## 1. Introduction

**Figure 1:**
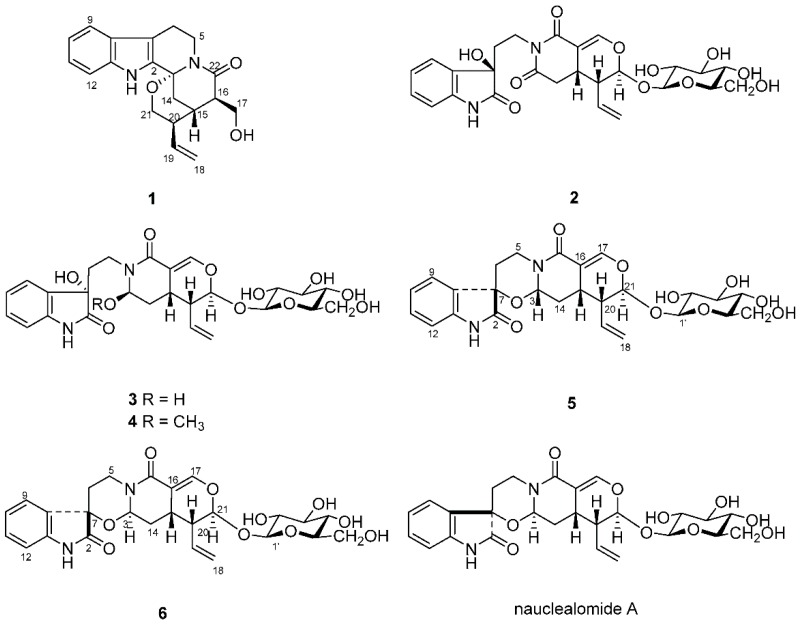
Structures of compounds **1**–**6** isolated from *N*. *officinalis*, as well as structure of nauclealomide A mentioned in the text.

*Nauclea officinalis* Pierre ex Pitard belongs to the genus *Nauclea* in family Rubiaceae, which is mainly distributed in Hainan, Guangxi, Guangdong and other provinces in the south of China. As a traditional Chinese medicine, the bark and twigs of *N*. *officinalis* is widely used for the treatment of colds, fever, acute tonsillitis, sore throat, conjunctivitis, enteritis, dysentery, eczema and other ailments [1]. Previous phytochemical investigation had revealed that monoterpene indole alkaloids, which present a polycyclic skeleton with a tetrahydro-β-carboline ring, are the main components of this plant [2,3,4,5,6,7,8,9,10,11,12,13]. The complex ring system and diversity of stereostructure of these monoterpene indole alkaloids have attracted great interest for phytochemical studies. As a part of our ongoing program to search for structurally unique alkaloids from the medicinal plants in southern China [5,13], we carried out the continuing investigation on the leaves of *N*. *officinalis*. In our present study, three new indole alkaloids, naucleamide G (**1**), and nauclealomide B and C (**5** and **6**), together with three known ones, paratunamide C (**2**), paratunamide D (**3**) and paratunamide A (**4**) (Figure 1), were isolated, among which oxindole alkaloids (**2**–**6**) were rarely reported from the *Nauclea* genus. In this paper, we describe the structure elucidation of these new alkaloids by means of NMR, HRESIMS, circular dichroism spectroscopy and computational analysis.

## 2. Results and Discussion

Compound **1** showed a molecular formula of C_20_H_22_N_2_O_3_ based on the HRESIMS (*m*/*z* 339.1703 [M + H]^+^; calculated for C_20_H_23_N_2_O_3_: *m*/*z* 339.1709). The UV spectrum of **1** showed absorption maxima at 211 and 290 nm. The IR spectrum exhibited absorptions at 3373 and 1636 cm^−1^, which suggested the presence of amino and α, β-unsaturated carbonyl functional groups. The ^1^H-NMR spectrum of **1** displayed four aromatic protons (δ_H_ 7.46 (1H, d, *J* = 8.0 Hz), 7.37 (1H, d, *J* = 8.0 Hz), 7.12 (1H, t, *J =* 8.0 Hz) and 7.00 (1H, t, *J =* 8.0 Hz)), three terminal vinyl protons (δ_H_ 6.39 (1H, ddd, *J =* 17.3, 10.4, 7.3 Hz), 5.28 (1H, d, *J =* 17.3 Hz) and 5.23 (1H, d, *J =* 10.4 Hz)) and one amino proton (δ_H_ 11.19 (1H, s)). The ^13^C-NMR and DEPT spectra of **1** displayed 20 signals, including six methylenes, eight methines, one carbonyl, and five quaternary carbons, suggesting the presence of a monoterpene indole alkaloid.

**Figure 2:**
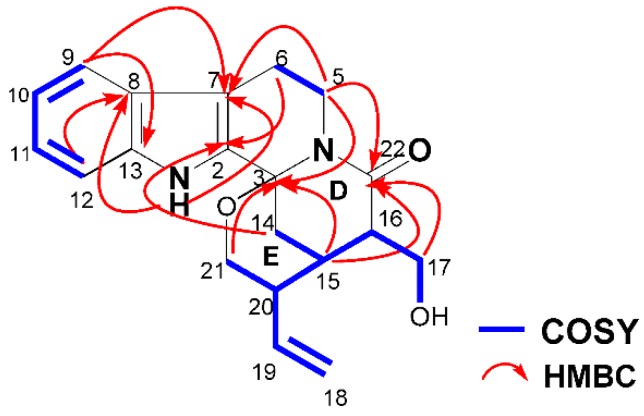
^1^H-^1^H COSY and key HMBC correlations of **1**.

With the aid of Two Dimensional ^1^H Correlation Spectroscopy (^1^H-^1^H COSY), ^1^H-Detected Heteronuclear Single Quantum Correlation (HSQC), 1H-Detected Heteronuclear Multiple Bond Correlation (HMBC), and Rotating-frame Overhauser Enhancement Spectroscopy (ROESY) experiments, all of the ^1^H and ^13^C-NMR signals of **1** were assigned as shown in Table 1. The ^1^H-^1^H COSY spectrum of **1** revealed the presence of three spin coupling systems (δ_H_ 7.46 (H-9)↔7.00 (H-10)↔7.12 (H-11)↔7.37 (H-12), δ_H_ 4.80 (Ha-5)↔2.61 (Hb-6) and δ_H_ 5.28 (Ha-18)/5.23 (Hb-18)↔6.39 (H-19)↔2.51 (H-20)↔2.33 (H-15)↔2.79 (H-16)↔4.08 (Ha-17)/3.65 (Hb-17)↔4.69 (H-OH), 2.33 (H-15)↔1.72 (Hb-14), 2.51 (H-20)↔3.59 (Hb-21)). In the HMBC spectrum, the correlations between H-1 (δ_H_ 11.19) and C-7 (δ_C_ 108.7)/C-8 (δ_C_ 125.3)/C-2 (δ_C_ 133.8)/C-13 (δ_C_ 136.2), between H-9 (δ_H_ 7.46) and C-7 (δ_C_ 108.7)/C-13 (δ_C_ 136.2), between H-12 (δ_H_ 7.37) and C-8 (δ_C_ 125.3), between Ha-5 (δ_H_ 4.80) and C-3 (δ_C_ 80.9)/C-7 (δ_C_ 108.7), as well as between Ha-6 (δ_H_ 2.74)/Hb-6 (δ_H_ 2.61) and C-2 (δ_C_ 133.8) revealed the presence of a tetrahydro-β-carboline ring. In addition, the correlations between Ha-5 (δ_H_ 4.80) and C-22 (δ_C_ 170.6), between Ha-14 (δ_H_ 2.89) and C-2 (δ_C_ 133.8), between Ha-21 (δ_H_ 3.66)/Hb-21 (δ_H_ 3.59)/Ha-14 (δ_H_ 2.89)/Hb-14 (δ_H_ 1.72)/H-15 (δ_H_ 2.33) and C-3 (δ_C_ 80.9), as well as between H-15 (δ_H_ 2.33)/Ha-17 (δ_H_ 4.08)/Hb-17 (δ_H_ 3.65) and C-22 (δ_C_ 170.6) revealed the presence of ring D, which was fused to the tetrahydro-β-carboline ring and ring E (Figure 2).

**Figure 3:**
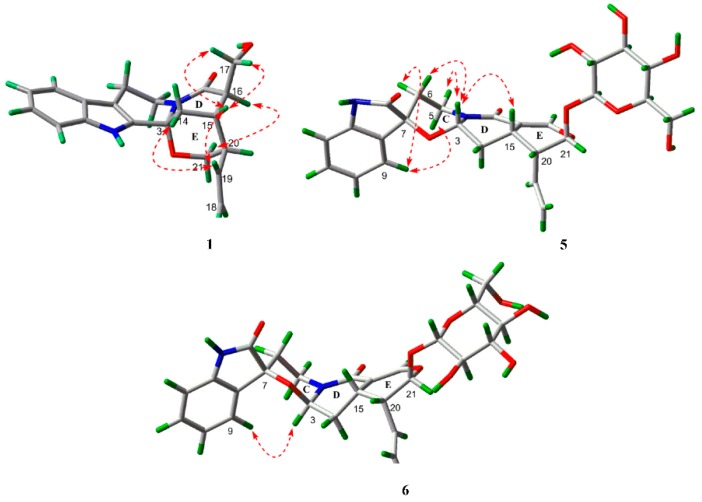
Selected ROESY correlations of **1**, **5** and **6**.

**Figure 4:**
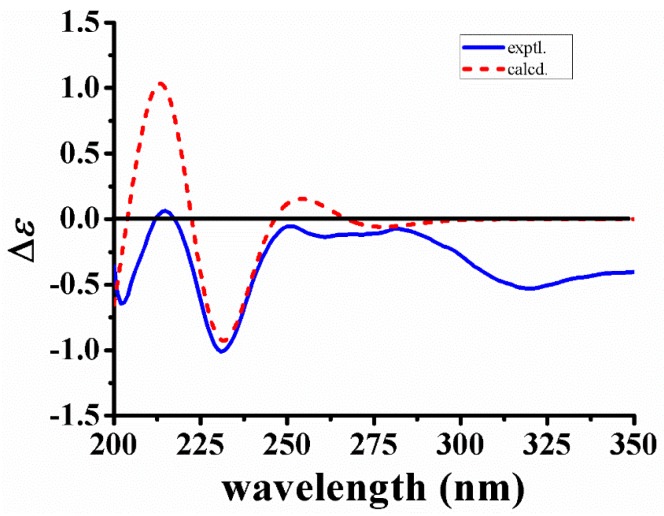
Calculated and experimental CD spectra of **1**.

The relative configuration of **1** was deduced from coupling constants and ROESY data. The coupling constants of ^3^*J*_14a,15_ *=* 3.3 Hz, ^3^*J*_14b,15_ = 2.0 Hz and ^3^*J*_15,16_ < 1.0 Hz, and the ROESY correlations between Hb-21 and H-16, as well as between H-15 and Ha-17/Hb-17 indicated both chair conformations of rings D and E, a cis-ring junction between rings D and E, and different orientations of H-15 and H-16 [14]. In addition, the ROESY correlations between H-19 and Ha-14/H-15 suggested different orientations of H-15 and H-20. On the basis of the above results, the relative configuration of **1** was elucidated as shown in Figure 3. The absolute configuration of **1** was confirmed by the quantum chemical CD calculation method at the (B3LYP/6-31G(d)) level using the GAUSSIAN 09 program, and the calculated CD curve of 3*R*,15*S*,16*R*,20*R*-**1** was similar to the measured spectrum (Figure 4). Therefore, the structure of **1** was established and named naucleamide G.

Compounds **5** and **6** showed the same molecular formula C_26_H_30_N_2_O_10_ as nauclealomide A by their HRESIMS (*m*/*z* 553.1782 [M + Na]^+^ and 553.1783 [M + Na]^+^). Moreover, the ^1^H and ^13^C-NMR data of **5** and **6** were similar to those of nauclealomide A (Table 2). Thus, the planar structures of **5**, **6** were identical to that of nauclealomide A (Figure 1) [13]. The coupling constants of ^3^*J*_20,21_ (*J* = 1.7 Hz) and ^3^*J*_20,15_ (*J* = 5.8 Hz) suggested the presence of β/β/α orientation for H-15, H-20 and H-21 in **5** and **6** [15,16,17].

**Scheme 1:**
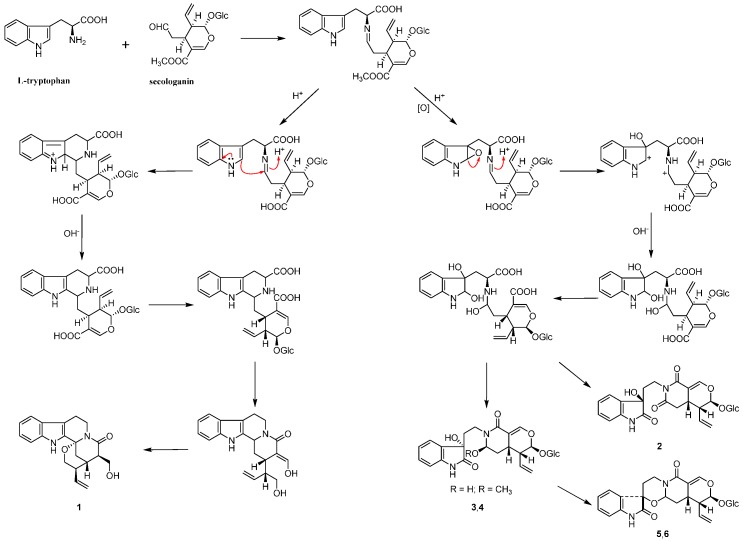
The plausible biosynthetic pathway for **1**–**6**.

In the ^1^H-NMR spectrum of **5**, the coupling constants of ^3^*J*_3,14a_ (*J* = 9.5 Hz) and ^3^*J*_3,14b_ (*J* = 4.6 Hz) indicated the presence of β configuration of H-3, which was confirmed by the ROESY correlation between H-3 and H-15. In the ROESY spectrum, the correlations between H-3 and Hb-5/Hb-6, as well as between H-9 and Ha-5/Ha-6 indicated the presence of α/β/α/β configuration of Ha-5, Hb-5, Ha-6 and Hb-6 (Figure 3). The absolute configuration of C-15 in these indole alkaloids was determined as *S* biosynthetically based on secologanin (Scheme 1). Thus, the absolute configuration of **5** was elucidated as 3*S*, 7*S*, 15*S*, 20*R*, 21*S*.

In the ^1^H-NMR spectrum of **6**, the coupling constants of ^3^*J*_3,14a_ (*J* = 3.6 Hz) and ^3^*J*_3,14b_ (*J* = 2.1 Hz) indicated the presence of *α* configuration of H-3. In the ROESY spectrum, the correlation between H-3 and H-9 was observed, which suggested that the carbonyl group at C-2 was above the C/D/E plane (Figure 3). Considering the biogenetic pathway for these indole alkaloids, the absolute configuration of C-15 was determined as *S*. Thus, the absolute configuration of **6** was elucidated as 3*R*, 7*S*, 15*S*, 20*R*, 21*S*.

The three known indole alkaloids were identified as paratunamide C (**2**) [18], paratunamide D (**3**) [18], and paratunamide A (**4**) [18] by comparison of their spectroscopic data with those reported in the literature.

Based on the structural features of compounds **1**–**6**, which were all derived from l-tryptophan and secologanin, the plausible biosynthetic pathway for them was deduced (Scheme 1).

## 3. Materials and Methods

### 3.1. General Procedures

Optical rotations were measured on a JASCO P-1020 polarimeter (JASCO Corporation, Tokyo, Japan). UV spectra were recorded on a JASCO V-550 UV/VIS spectrophotometer (JASCO Corporation). A JASCO FT/IR-480 plus FT-IR spectrometer (JASCO Corporation) was used for scanning the IR spectra with KBr pellets. ^1^H, ^13^C, and 2D NMR spectra were recorded on a Bruker AV-400 spectrometer (Bruker Corporation, Rheinstetten, Germany). HR-ESI-MS data were conducted on an Agilent 6210 LC/MSD TOF mass spectrometer. CD spectra were obtained using a JASCO J-810 circular dichroism spectrometer. For column chromatography, silica gel (300–400 mesh; Qingdao Marine Chemical Group Corporation, Qingdao, China) and Sephadex LH-20 (Pharmacia, Pittsburgh, PA, USA) were used. TLC analyses were carried out using precoated silica gel GF_254_ plates (Yantai Chemical Industry Research Institute, Yantai, China). Analytic high-performance liquid chromatography (HPLC) was performed on a SHIMADZU chromatography (SHIMADZU Corporation, Kyoto, Japan) equipped with an LC-20AD pump and an SPD-M20A diode-array detector (DAD) with an Inertsil ODS-3 column (4.6 mm × 250 mm, 5 μm). Preparative HPLC was carried out on a SHIMADZU instrument equipped with an LC-20AP pump and an SPD-20A (SHIMADZU Corporation) detector with a YMC-Pack ODS-A column (20 mm × 250 mm, 5 μm).

### 3.2. Plant Material

The leaves of *N. officinalis* were collected in Sanya City, Hainan Province, China, in July 2008, and authenticated by Professor Wei-ping Chen (Hainan Branch Institute of Medicinal Plants, Chinese Academy of Medical Science, Haikou, China). A voucher specimen (No. 20090223) was deposited in the Institute of Traditional Chinese Medicine and Natural Products, Jinan University, Guangzhou, China.

### 3.3. Extraction and Isolation 

Air-dried and powdered leaves of *N*. *officinalis* (4.8 kg) were extracted with 95% EtOH (50 L × 3, 3 days each) at room temperature. The EtOH solution was evaporated under reduced pressure to afford a residue (250.0 g), which was suspended in water and partitioned successively with light petroleum, EtOAc and *n*-BuOH. The *n*-BuOH fraction (60.0 g) was subjected to silica gel CC, eluting with CHCl_3_-MeOH (100:0→0:100) to give nine fractions (Fr. 1–9). Fr. 1 (5.0 g) was separated by a silica gel column with CHCl_3_-MeOH (95:5) as the eluent to give fifteen subfractions (Fr. 1-1–Fr. 1-15). Fr. 1–2 (90.0 mg) was subsequently purified by a Sephadex LH-20 column eluted with MeOH to yield compounds **1** (5.0 mg). Fr. 7 (1.0 g) was further purified on a Sephadex LH-20 column using MeOH as eluent, then compounds **2** (22.0 mg), **3** (32.0 mg), **4** (17.0 mg), **5** (12.0 mg) and **6** (8.0 mg) were finally obtained by preparative HPLC using MeOH-H_2_O (40:60, 8 mL/min) as the mobile phase.

### 3.4. Compound Characterization

*Naucleamide G* (**1**): orange amorphous powder; ${\left[\alpha \right]}_{D}^{25}$ −28.8 (*c* 0.18, MeOH); UV (MeOH) λ_max_ (log *ε*): 211 (4.11), 290 (3.52) nm; IR (KBr): *ν*_max_ 3373, 1692, 1636, 1409, 1174 cm^−1^; HR-ESI-MS *m*/*z*: 339.1703 [M + H]^+^ (calcd for C_20_H_23_N_2_O_3_, 339.1709); ^1^H-NMR (DMSO-*d*_6_, 400 MHz) and ^13^C-NMR (DMSO-*d*_6_, 100 MHz), see Table 1 and Supplementary Materials.

*Nauclealomide B* (**5**): yellowish amorphous solid; ${\left[\alpha \right]}_{D}^{27}$ −269 (*c* 0.25, MeOH); UV (MeOH) λ_max_(log *ε*): 211 (4.31), 243 (4.12) nm; IR (KBr) *ν*_max_: 3417, 1713, 1657, 1473, 1067 cm^−1^; HR-ESI-MS *m*/*z*: 553.1782 [M + Na]^+^ (calcd for C_26_H_30_N_2_O_10_Na, 553.1793); ^1^H-NMR (CD_3_OD, 400 MHz) and ^13^C-NMR (CD_3_OD, 100 MHz), see Table 2.

*Nauclealomide C* (**6**): yellowish amorphous solid; ${\left[\alpha \right]}_{D}^{27}$ −117 (*c* 0.17, MeOH); UV (MeOH) λ_max_ (log *ε*): 212 (4.33), 242 (4.10) nm; IR (KBr) *ν*_max_: 3404, 1733, 1669, 1475, 1061 cm^−1^; HR-ESI-MS *m*/*z*: 553.1783 [M + Na]^+^ (calcd for C_26_H_30_N_2_O_10_Na, 553.1793); ^1^H-NMR (CD_3_OD, 400 MHz) and ^13^C-NMR (CD_3_OD, 100 MHz), see Table 2.

### 3.5. Acid Hydrolysis and Sugar Analysis

Each solution of compounds **5** and **6** (2 mg each) in 2 mol/L HCl (5 mL) was hydrolyzed at 80 °C for 2 h. The solution was then evaporated under reduced pressure. The residue was dissolved in 1 mL of pyridine containing 2 mg of l-cysteine methyl ester hydrochloride, after which the reaction mixture was heated at 60 °C for 1 h. *O*-tolyl isothiocyanate (5 μL) was then added to the mixture and heated at 60 °C for an additional 1 h. The reaction mixture was subsequently analyzed by HPLC and detected at 250 nm. Analytical HPLC was performed on an Inertsil ODS-3 column (4.6 × 250 mm, 5 μm) at 20 °C using CH_3_CN-0.05% CH_3_COOH in H_2_O (25:75, 1.0 mL/min) as the mobile phase. Peaks were detected with an SPD-M20A DAD. D-Glucose (*t*_R_ 16.4 min) was identified as the sugar moiety of indole alkaloids **5** and **6** by comparison with authentic samples of d-glucose (*t*_R_ 16.4 min) and l-glucose (*t*_R_ 14.9 min) [19].

## 4. Conclusions

Six indole alkaloids, including three new ones **1**, **5** and **6**, named naucleamide G, and nauclealomide B and C, were isolated from the *n*-BuOH-soluble fraction of an EtOH extract of the leaves of *N*. *officinalis*. The structures and absolute configurations of the new indole alkaloids were elucidated by means of NMR, HRESIMS, acid hydrolysis and quantum chemical CD calculation. Compounds **2**–**4** were isolated from the *Nauclea* genus for the first time and oxindole alkaloids (**2**–**6**) were rare in this genus. Thus, the findings complemented previous reports of the occurrence of indole alkaloids in the *Nauclea* genus and might be a useful chemotaxonomic marker of this genus. In addition, a plausible biosynthetic pathway for all the alkaloids was proposed. Compound **1** with a tetrahydro-β-carboline ring originated from condensation of l-tryptophan with secologanin to give strictosidine; nevertheless, oxindole alkaloids (**2**–**6**) might be generated through another biosynthetic route.

## Figures and Tables

**Table 1:** ^1^H (400 MHz) and ^13^C (100 MHz) NMR data of **1** (dimethylsulfoxide-*d*_6_ (DMSO-*d*_6_), δ in ppm, *J* in Hz).

Position	δ_C_	δ_H_
1	-	11.19 s
2	133.8	-
3	80.9	-
5	34.6	a 4.80 dd (12.7, 4.7)
		b 2.82 m
6	20.7	a 2.74 m
		b 2.61 m
7	108.7	-
8	125.3	-
9	118.6	7.46 d (8.0)
10	118.7	7.00 t (8.0)
11	121.9	7.12 t (8.0)
12	111.5	7.37 d (8.0)
13	136.2	-
14	31.5	a 2.89 dd (13.0, 3.3)
		b 1.72 dd (13.0, 2.0)
15	30.9	2.33 br s
16	46.8	2.79 m
17	58.9	a 4.08 m
		b 3.65 m
17-OH	-	4.69 dd (6.0, 4.3)
18	115.5	a 5.28 d (17.3)
		b 5.23 d (10.4)
19	140.4	6.39 ddd (17.3, 10.4, 7.3)
20	35.8	2.51 m
21	61.7	a 3.66 m
		b 3.59 dd (12.7, 3.1)
22	170.6	-

**Table 2:** ^1^H (400 MHz) and ^13^C (100 MHz) NMR data of nauclealomide A, **5** and **6** (CD_3_OD, δ in ppm, *J* in Hz).

Position	Nauclealomide A		5		6	
	δ_C_	δ_H_	δ_C_	δ_H_	δ_C_	δ_H_
2	180.1	-	179.5	-	177.8	-
3	80.0	6.28 t (2.8)	80.4	6.04 dd (9.5, 4.6)	81.2	5.72 dd (3.6, 2.1)
5	39.3	a 4.60 ddd (13.0, 4.9, 1.8)	36.1	a 4.43 dt (13.3, 4.7)	39.7	a 4.73 ddd (13.6, 5.4, 2.8)
		b 3.75 dd (13.0, 3.0)		b 3.73 ddd (13.3, 11.0, 3.7)		b 3.55 td (13.6, 3.3)
6	32.4	a 2.11 td (14.1, 5.4)	31.7	a 2.16 ddd (14.2, 11.0, 4.9)	31.3	a 2.17 td (13.3, 5.6)
		b 1.77 m		b 2.03 dt (14.2, 4.0)		b 1.67 m
7	77.6	-	76.3	-	79.4	-
8	131.4	-	131.5	-	131.6	-
9	125.4	7.25 br d (7.5)	125.1	7.27 br d (7.7)	126.6	7.82 br d (7.6)
10	123.9	7.02 br t (7.5)	123.9	7.00 td (7.7, 1.1)	123.7	7.07 td (7.6, 0.9)
11	131.1	7.25 br t (7.5)	131.2	7.24 td (7.7, 1.1)	131.1	7.31 td (7.6, 0.9)
12	111.0	6.84 br d (7.5)	111.2	6.85 br d (7.7)	111.9	6.94 br d (7.6)
13	142.3	-	142.4	-	142.6	-
14	29.7	1.76 m	32.2	a 1.45 dt (13.9, 9.5)	29.9	a 1.78 td (14.0, 3.6)
				b 1.95 dt (12.9, 4.6)		b 1.71 m
15	23.4	3.27 m	24.4	3.02 m	23.5	3.34 m
16	109.0	-	109.1	-	109.2	-
17	149.7	7.44 d (2.4)	149.1	7.40 d (2.3)	149.5	7.42 d (2.4)
18	120.7	a 5.29 dd (12.1, 1.6)	120.7	5.24 dd (14.5, 1.6)	120.6	5.22 m
		b 5.24 td (5.2, 1.8)		5.21 dd (7.8, 1.8)		
19	134.1	5.56 dt (17.2, 10.1)	133.8	5.53 dt (17.2, 10.0)	134.0	5.54 dt (17.6, 9.8)
20	44.3	2.61 ddd (10.1, 5.8, 1.7)	44.4	2.60 ddd (10.0, 5.8, 1.7)	44.2	2.56 ddd (9.8, 5.8, 1.7)
21	98.1	5.46 d (1.7)	97.3	5.47 d (1.7)	97.6	5.45 d (1.7)
22	166.1	-	165.7	-	166.1	-
1’	100.4	4.69 d (7.9)	99.6	4.65 d (7.9)	100.0	4.66 d (7.8)
2’	74.7	3.20 m	74.6	3.18 dd (8.9, 7.9)	74.6	3.24 dd (9.0, 7.8)
3’	78.2	3.37 m	77.8	3.36 m	77.7	3.39 m
4’	71.5	3.30 m	71.5	3.30 m	71.5	3.31 m
5’	78.3	3.31 m	78.2	3.31 m	78.3	3.31 m
6’	62.6	a 3.87 dd (11.8, 1.7)	62.6	a 3.87 dd (11.9, 1.6)	62.7	a 3.88 dd (11.9, 1.6)
		b 3.66 dd (11.8, 5.3)		b 3.66 dd (11.9, 5.4)		b 3.67 dd (11.9, 5.3)

## Supplementary Materials

The supplementary materials are available online at www.mdpi.com/1420-3049/21/8/968/s1, HRESIMS, UV, IR, and NMR spectra of indole alkaloids **1**, **5** and **6**.
