# Supplementary material for: Indole Alkaloids from the Leaves of Nauclea officinalis
Source: Molecules. 2016 Jul 23;21(8):968. doi: 10.3390/molecules21080968 (PMC6273496; doi:10.3390/molecules21080968)
Supplement: Supplementary file 1 [file molecules-21-00968-s001.pdf]

Molecules **2016**, *21*, 968; doi:10.3390/molecules21080968

Supplementary Materials: Indole Alkaloids from the Leaves of *Nauclea officinalis*

Long Fan, Cheng-Hui Liao, Qiang-Rong Kang, Kai Zheng, Ying-Chun Jiang and Zhen-Dan He

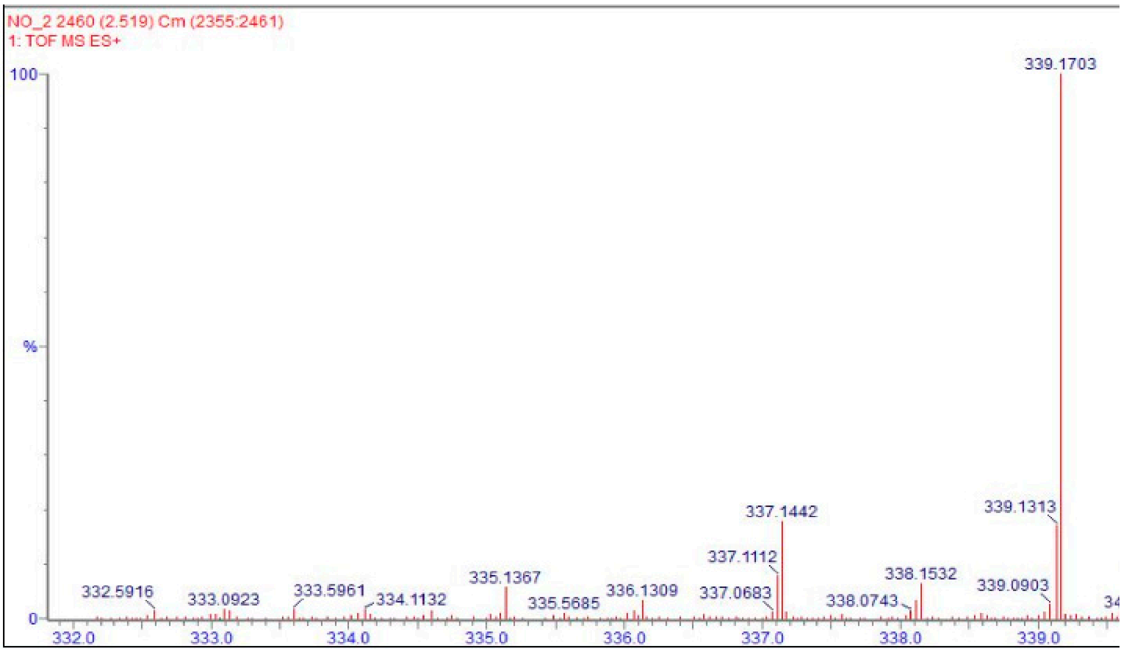

Figure S1. HR-ESI-MS spectrum of **1**.

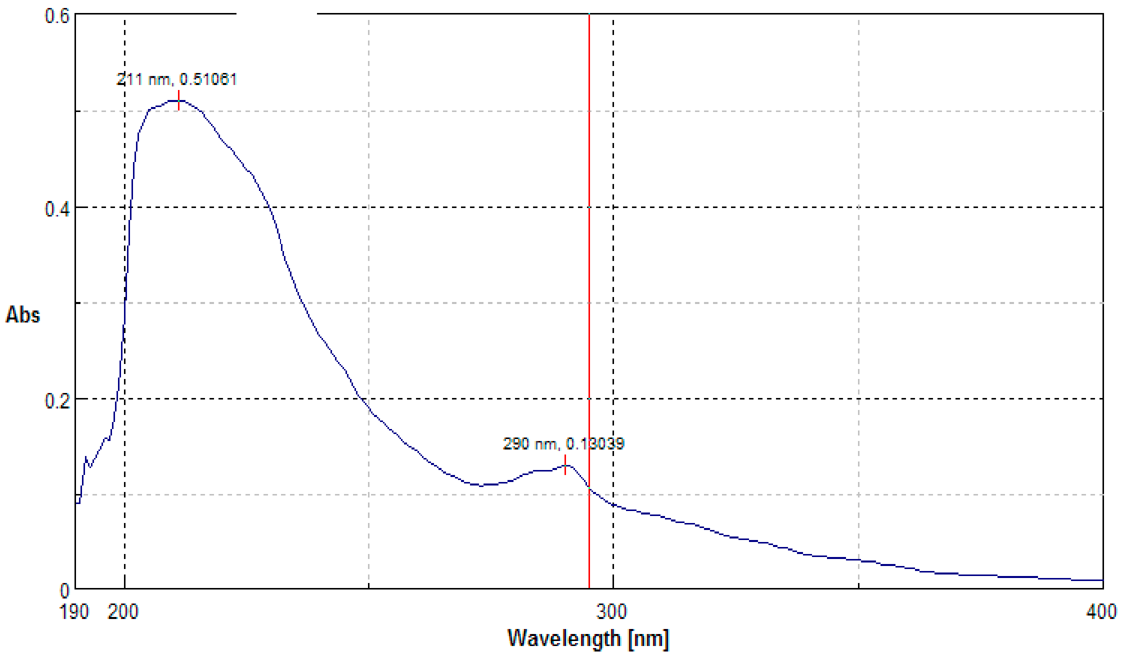

Figure S2. UV spectrum of **1** in MeOH.

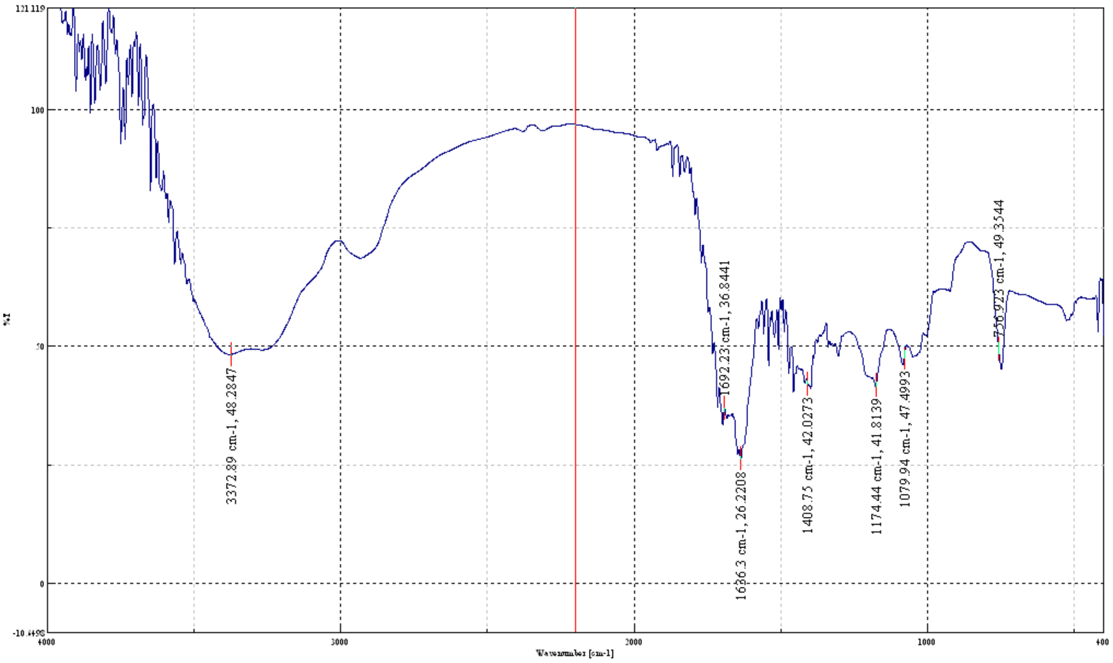

Figure S3. IR (KBr disc) spectrum of **1**.

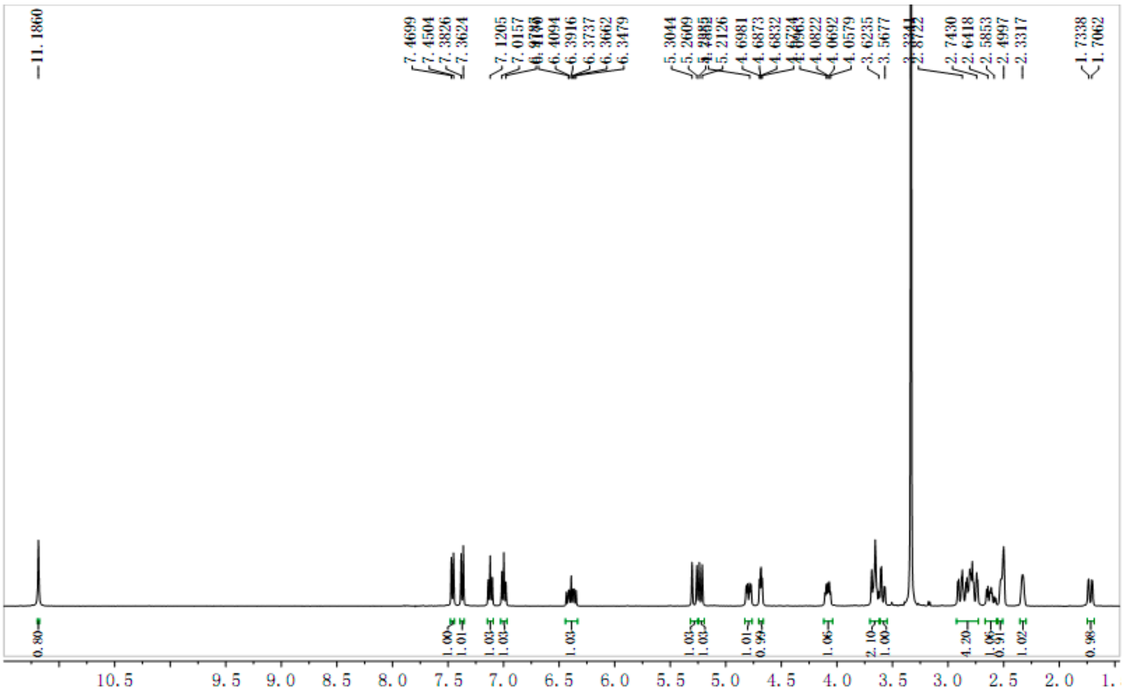

Figure S4. ^{1}H-NMR spectrum of **1** in DMSO-d_6.

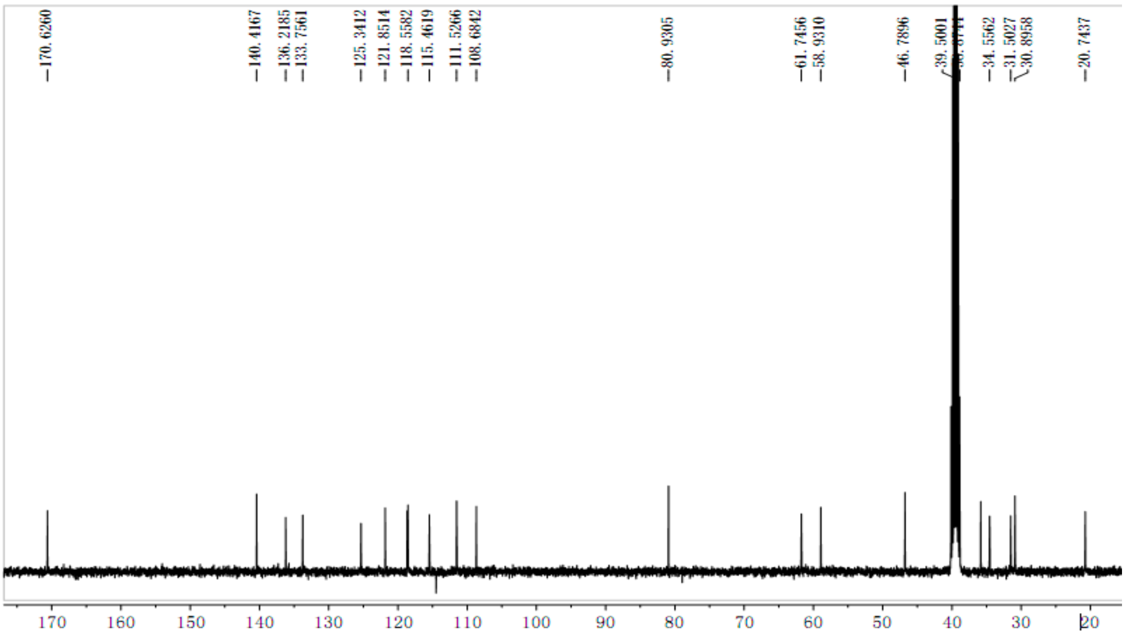

Figure S5. ^{13}C-NMR spectrum of **1** in DMSO-*d*$_6$.

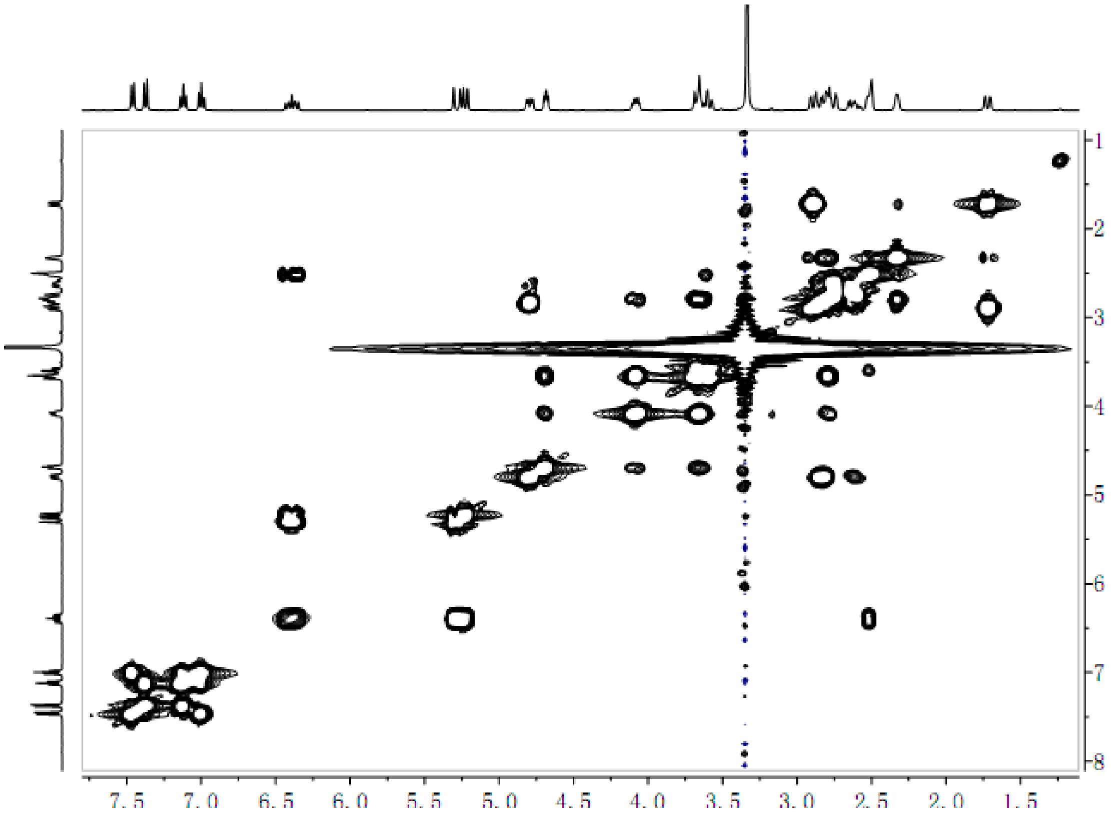

Figure S6. ^{1}H-^{1}H COSY spectrum of **1** in DMSO-*d*$_6$.

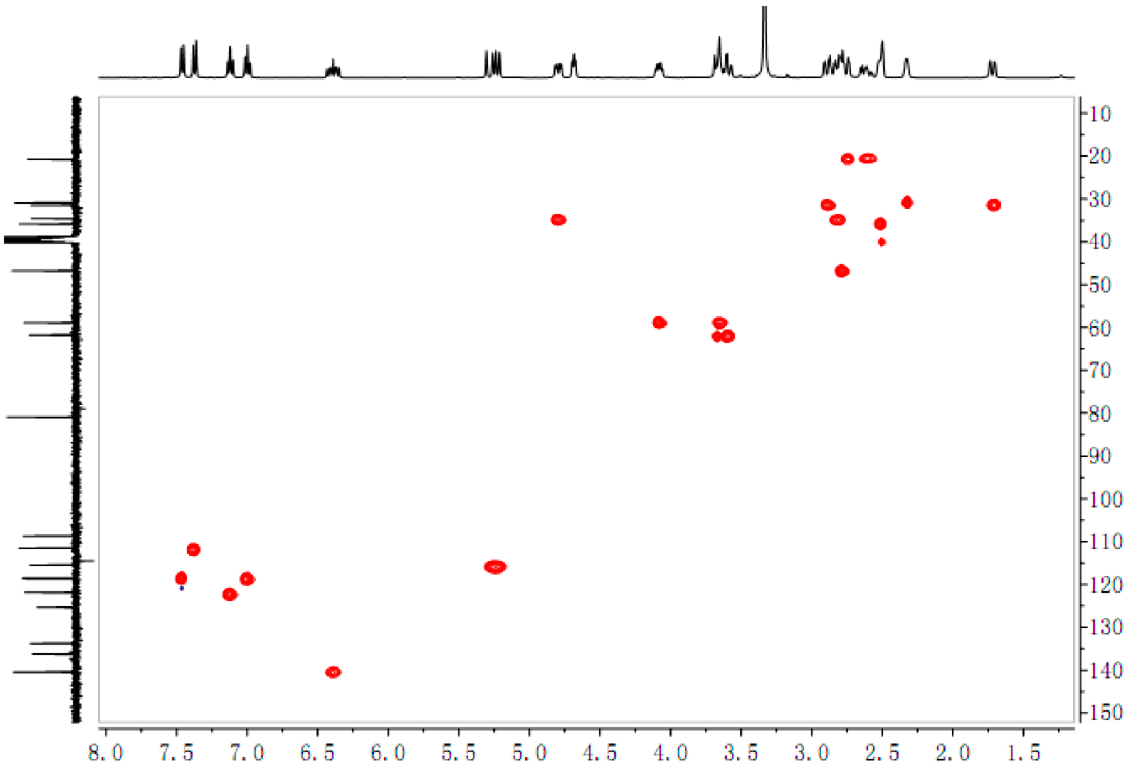

Figure S7. HSQC spectrum of **1** in DMSO-*d*6.

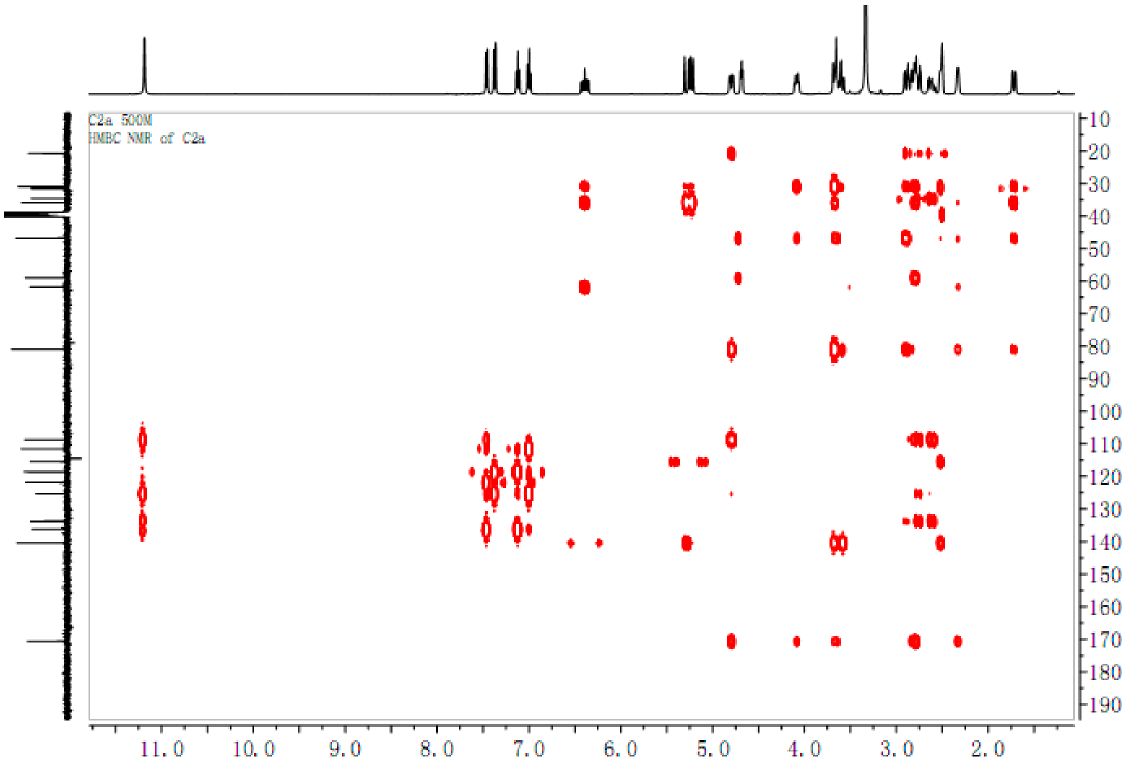

Figure S8. HMBC spectrum of **1** in DMSO-*d*6.

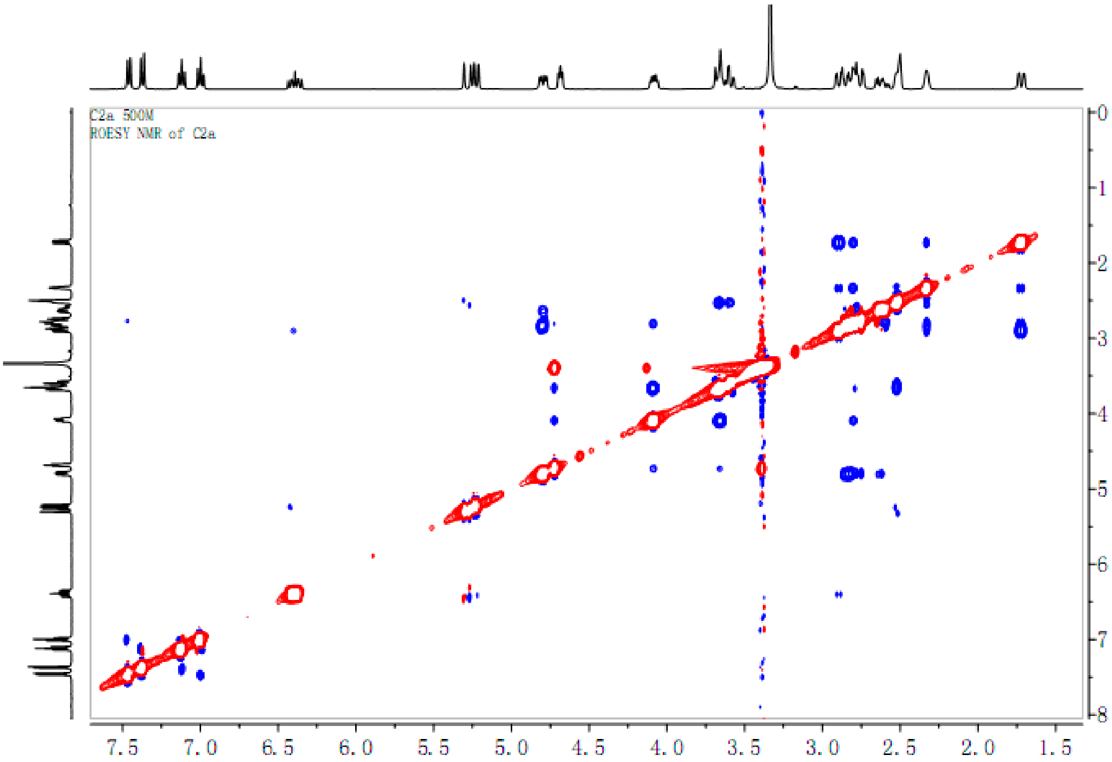

Figure S9. ROESY spectrum of **1** in DMSO-d_6.

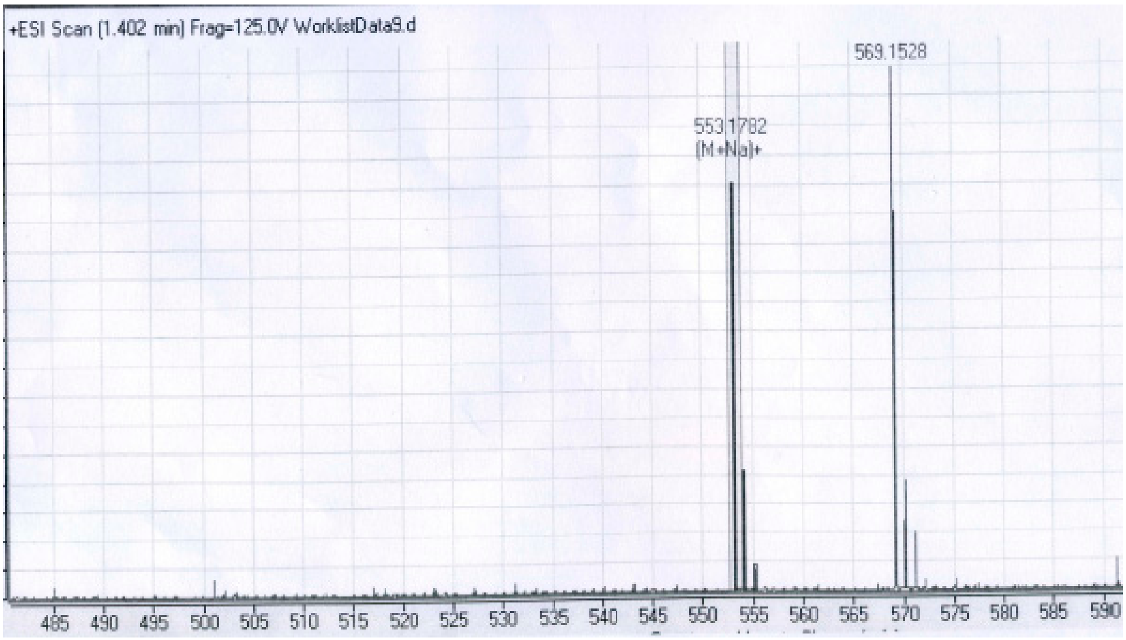

Figure S10. HR-ESI-MS spectrum of **5**.

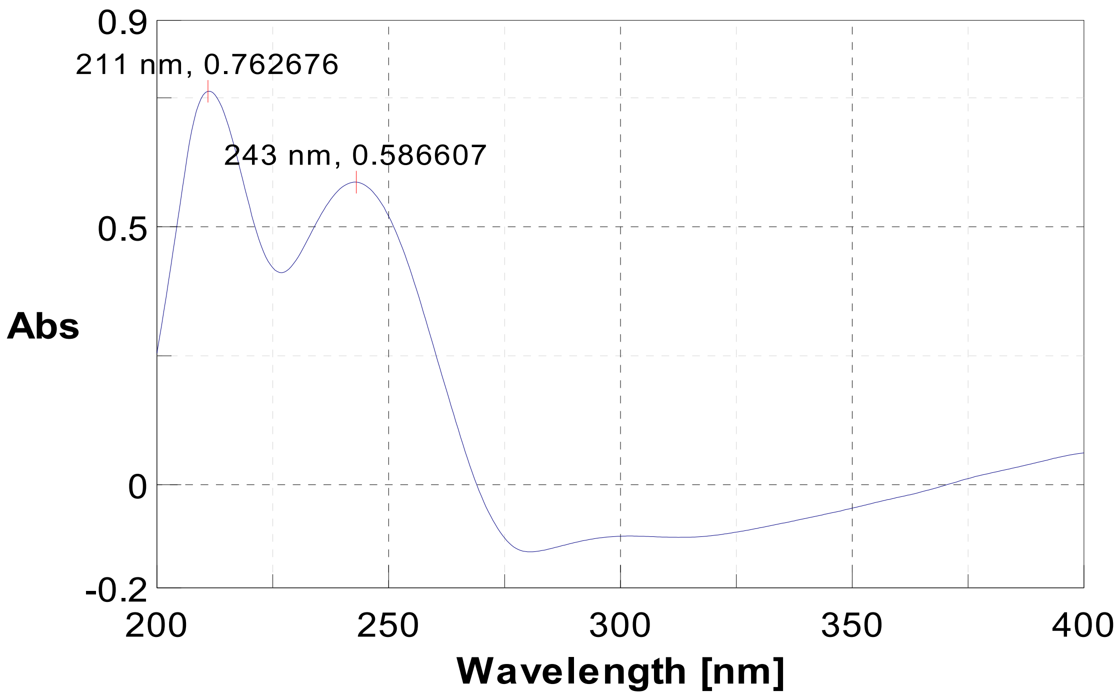

Figure S11. UV spectrum of **5** in MeOH.

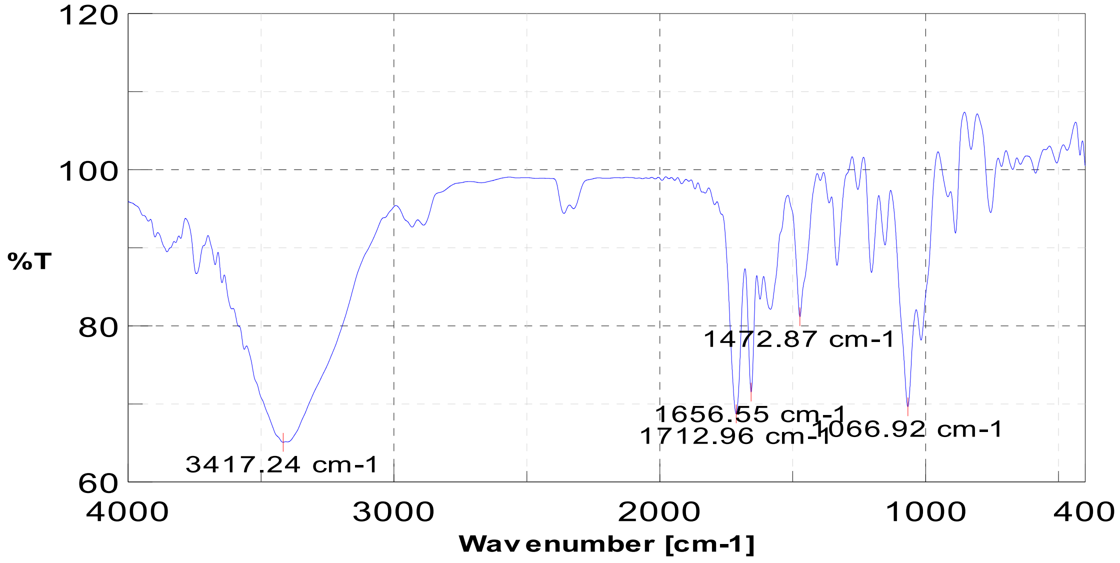

Figure S12. IR (KBr disc) spectrum of **5**.

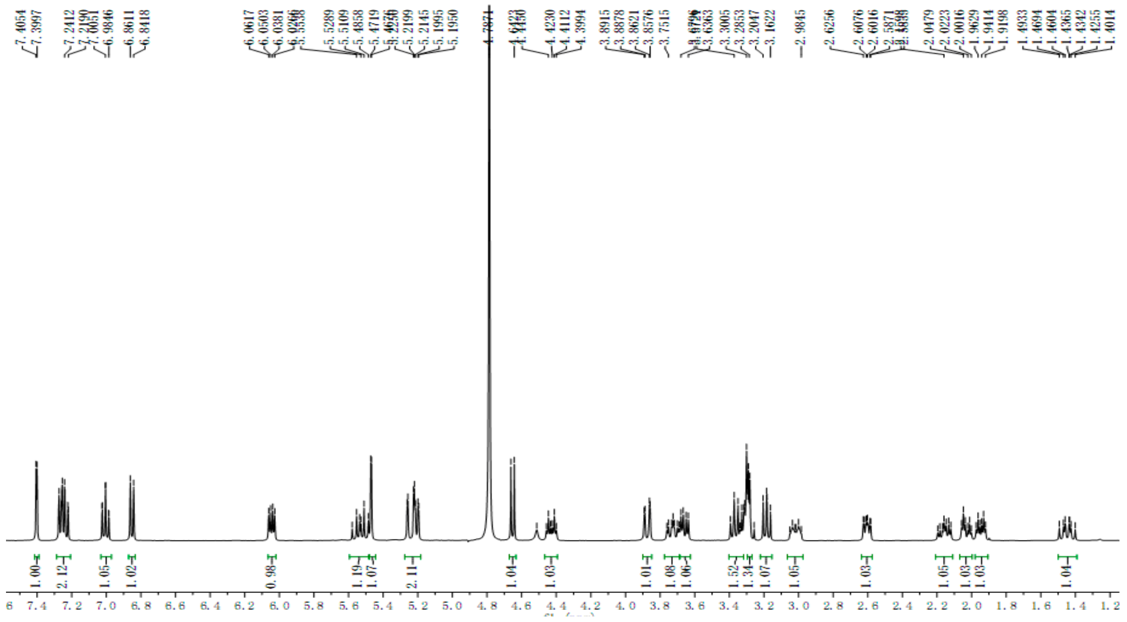

Figure S13. ^{1}H-NMR spectrum of **5** in MeOD.

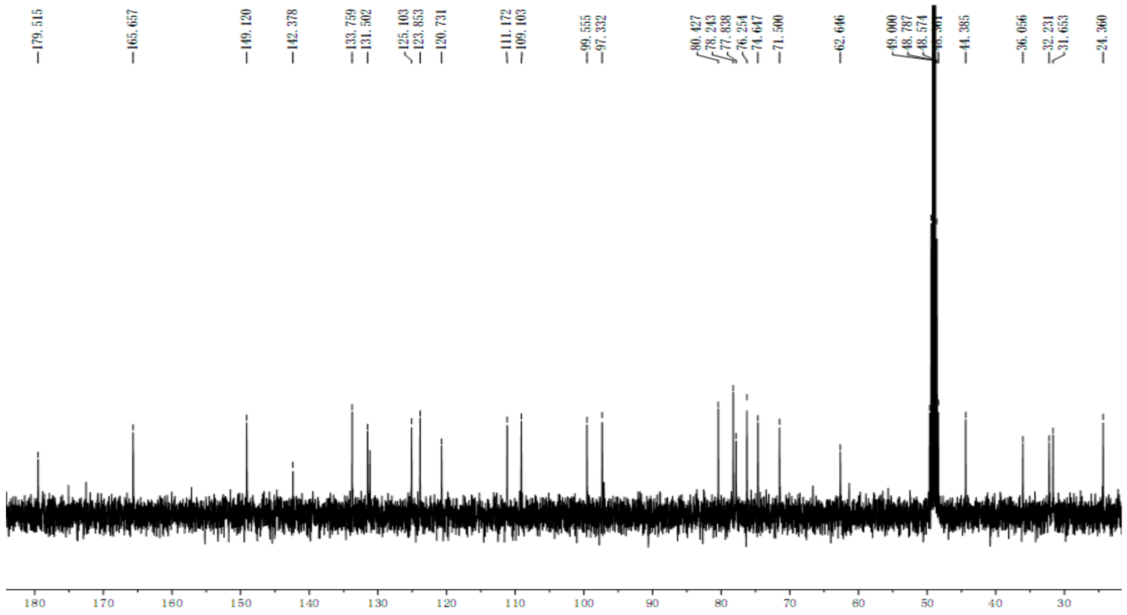

Figure S14. ^{13}C-NMR spectrum of **5** in MeOD.

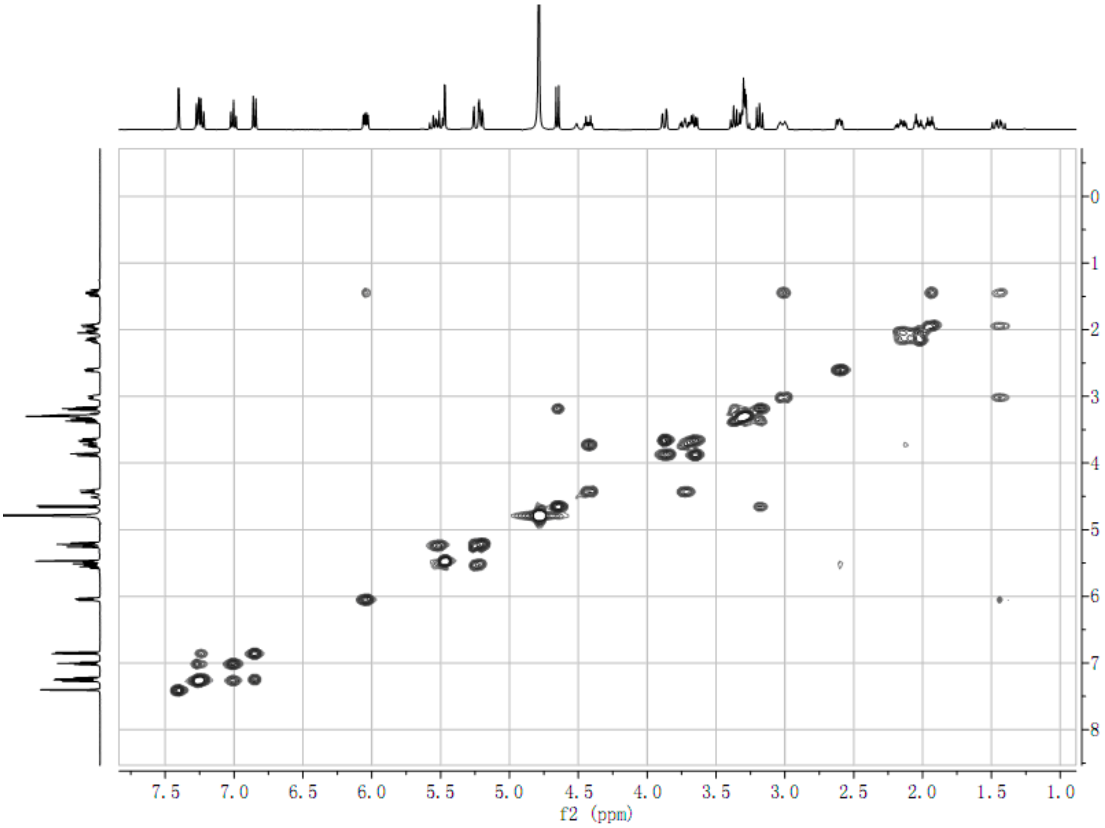

Figure S15. ^{1}H-^{1}H COSY spectrum of **5** in MeOD.

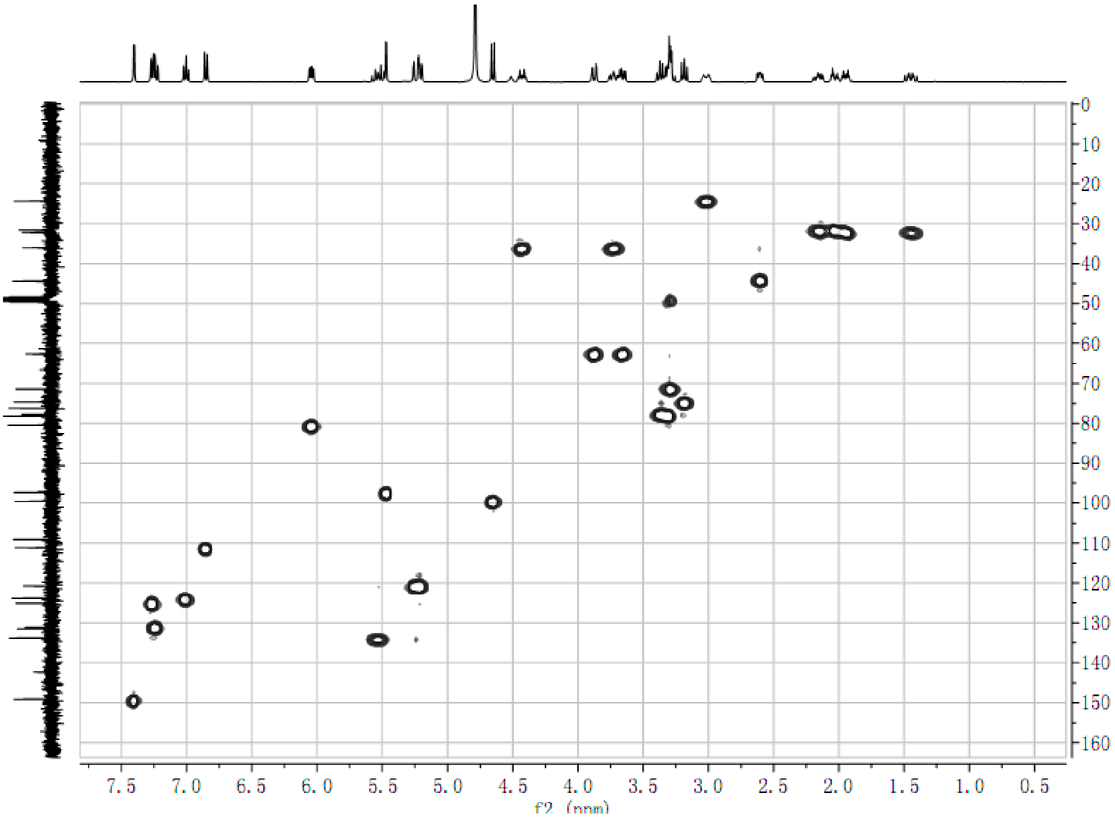

Figure S16. HSQC spectrum of **5** in MeOD.

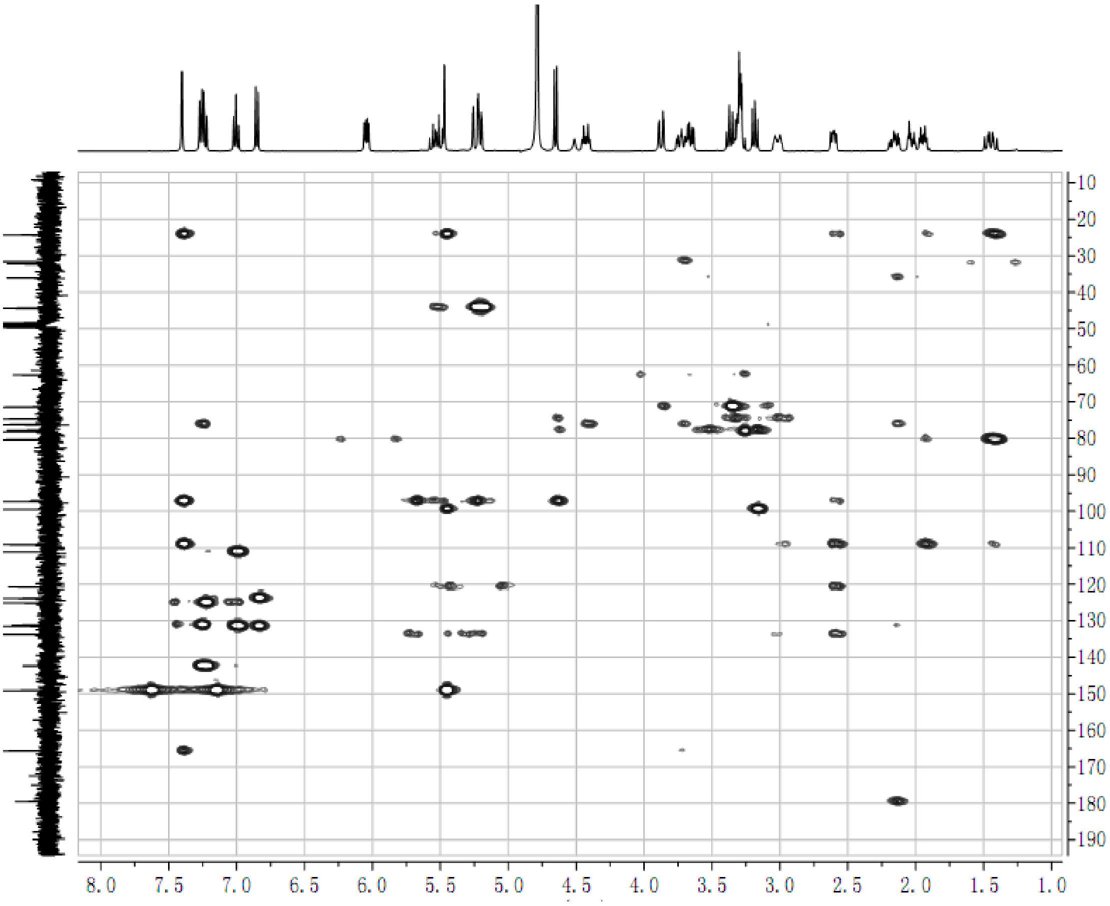

Figure S17. HMBC spectrum of **5** in MeOD.

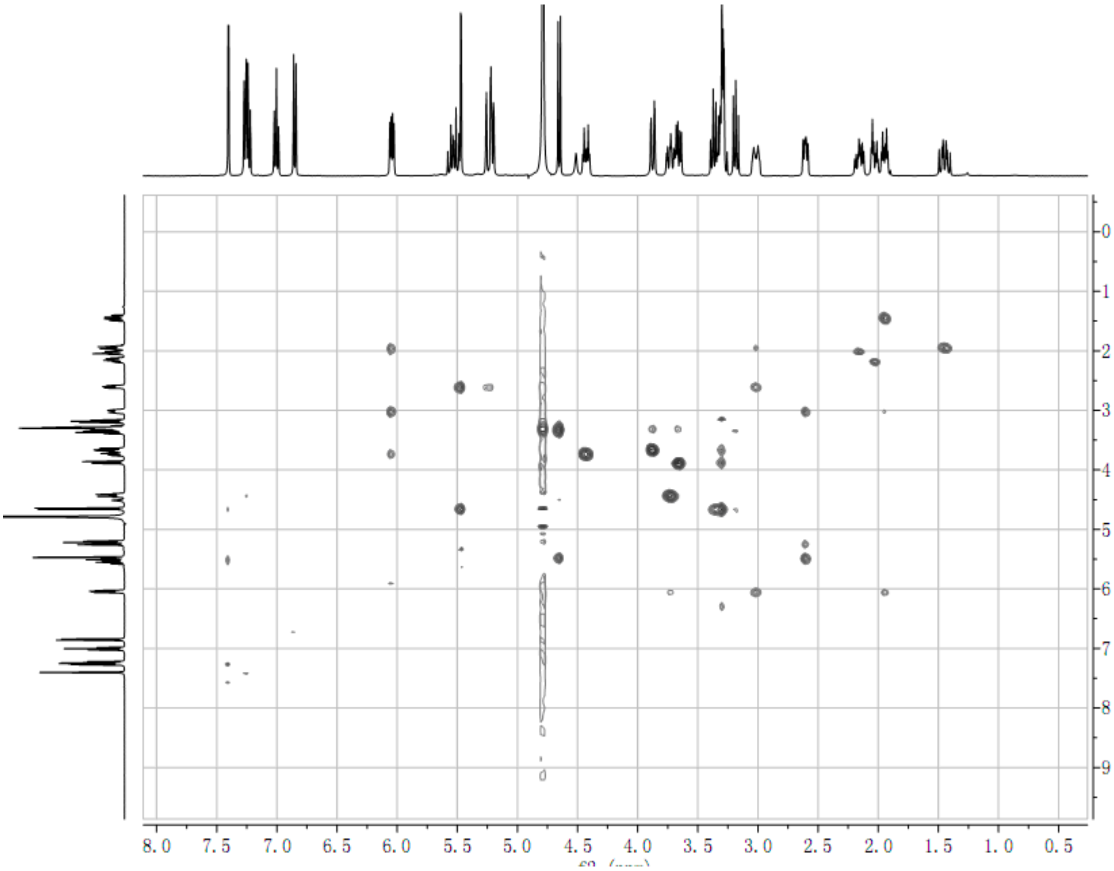

Figure S18. ROESY spectrum of **5** in MeOD.

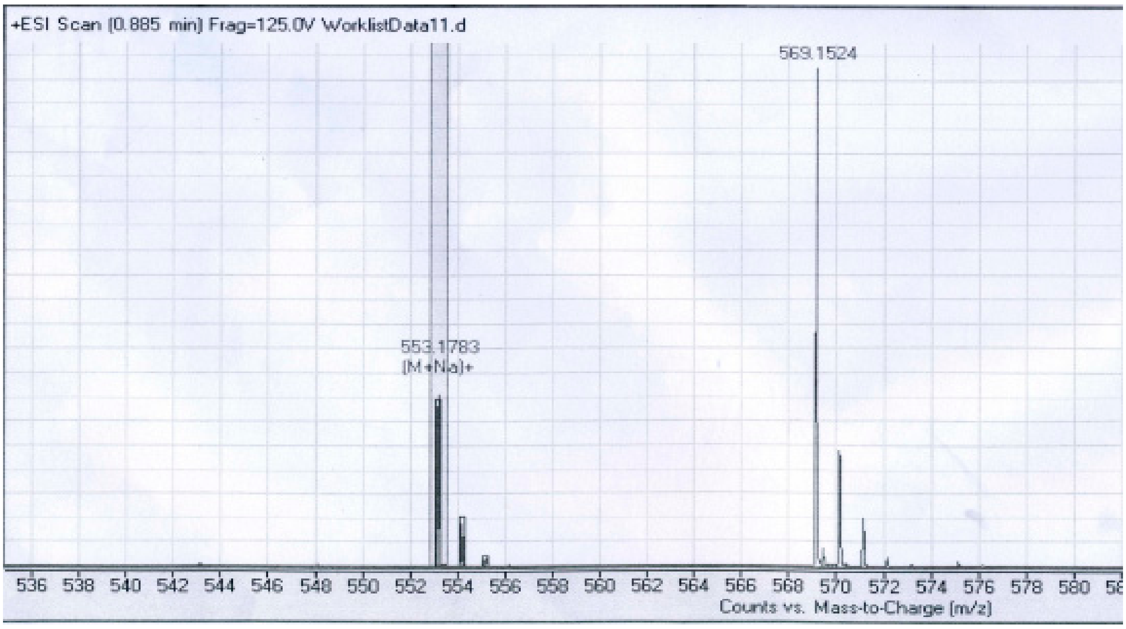

Figure S19. HR-ESI-MS spectrum of **6**.

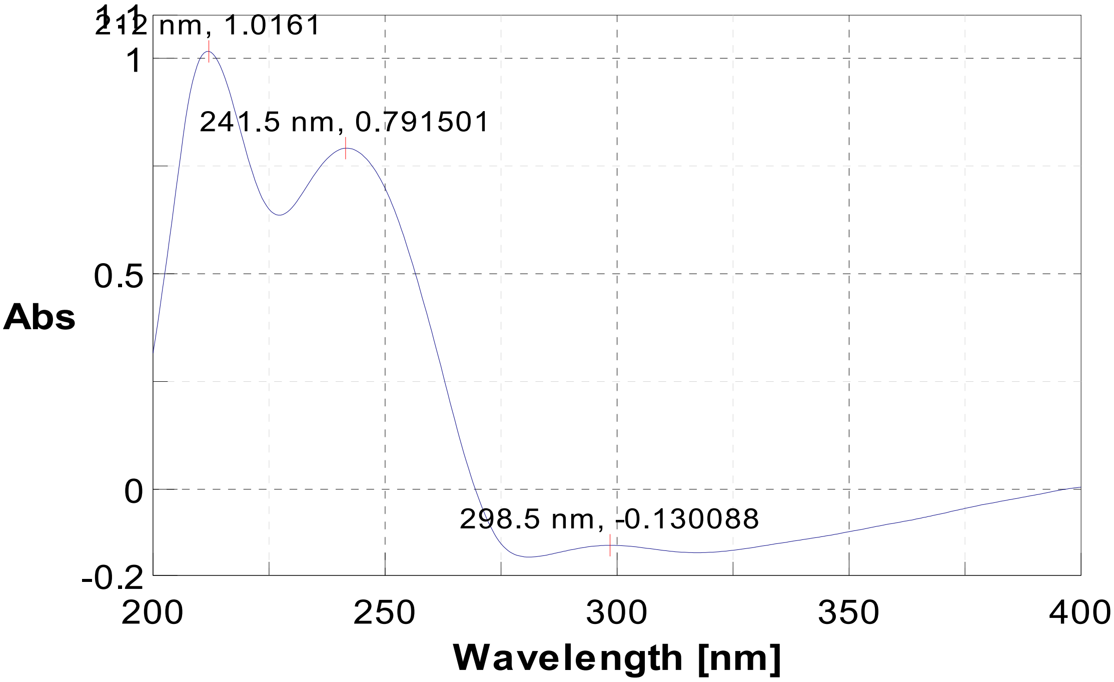

Figure S20. UV spectrum of **6** in MeOH.

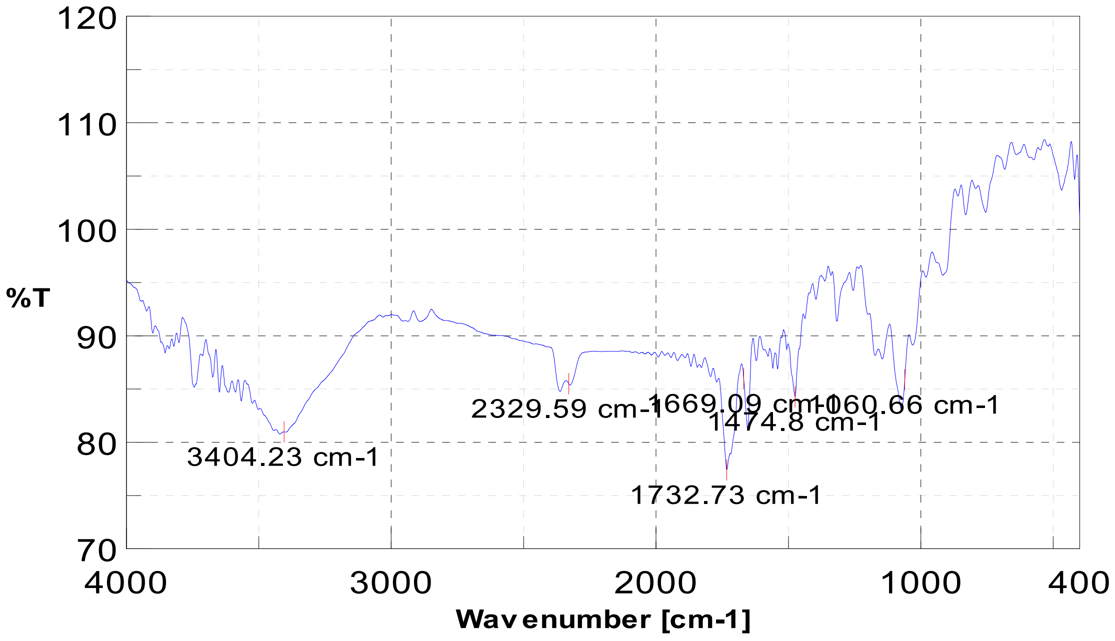

Figure S21. IR (KBr disc) spectrum of **6**.

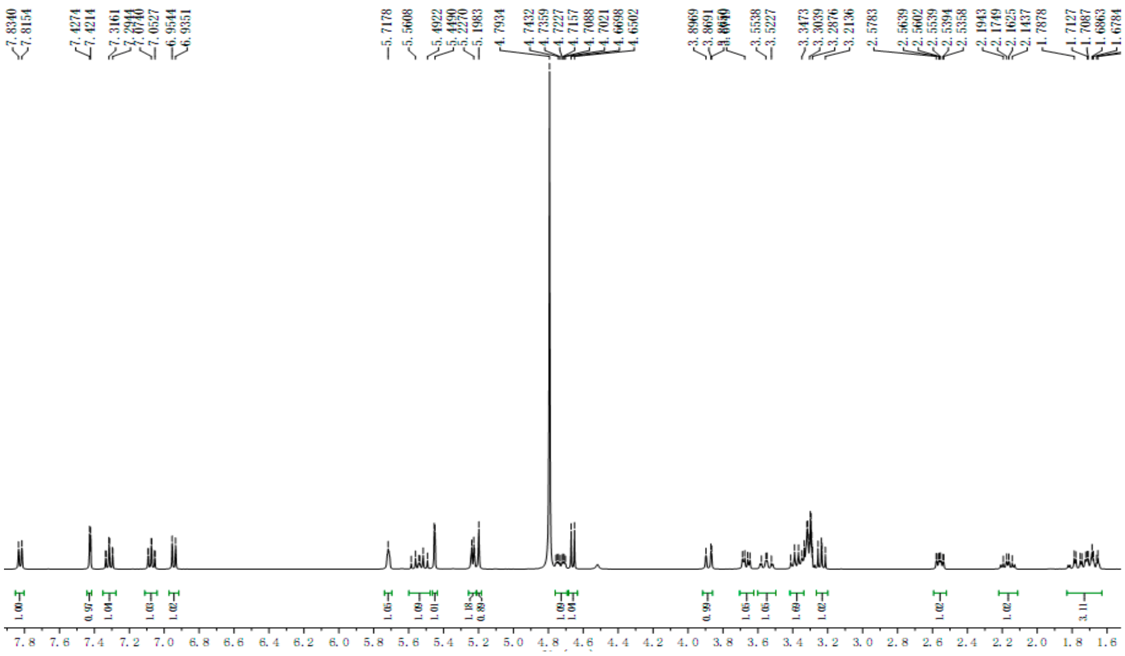

Figure S22. ^{1}H-NMR spectrum of **6** in MeOD.

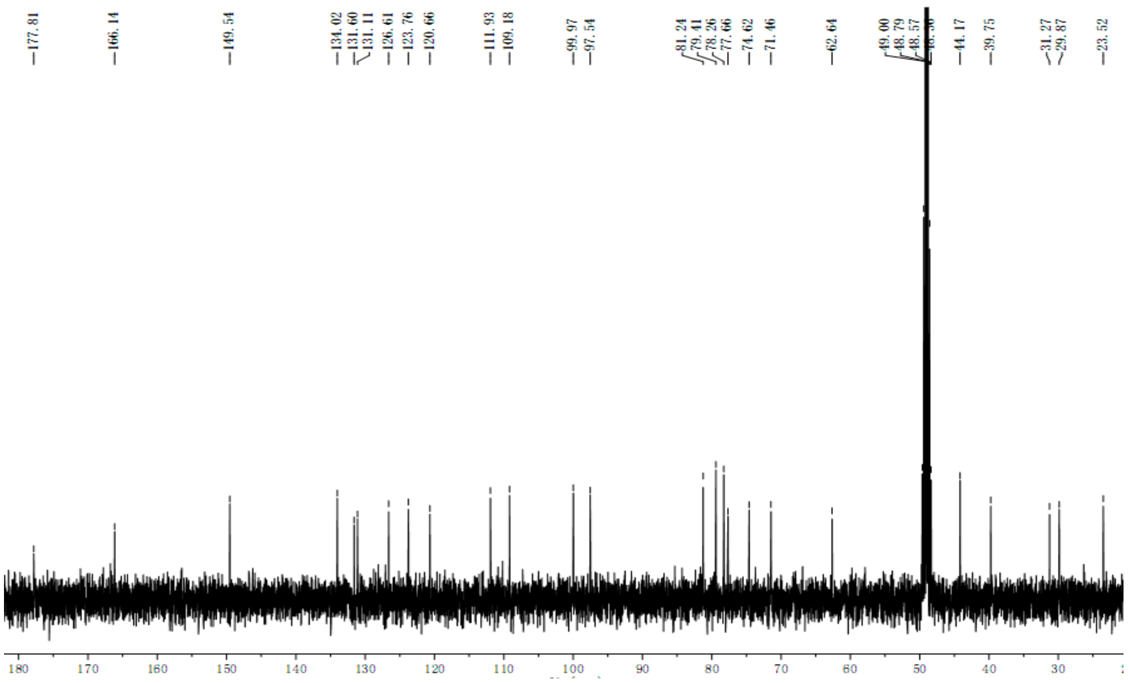

Figure S23. ^{13}C-NMR spectrum of **6** in MeOD.

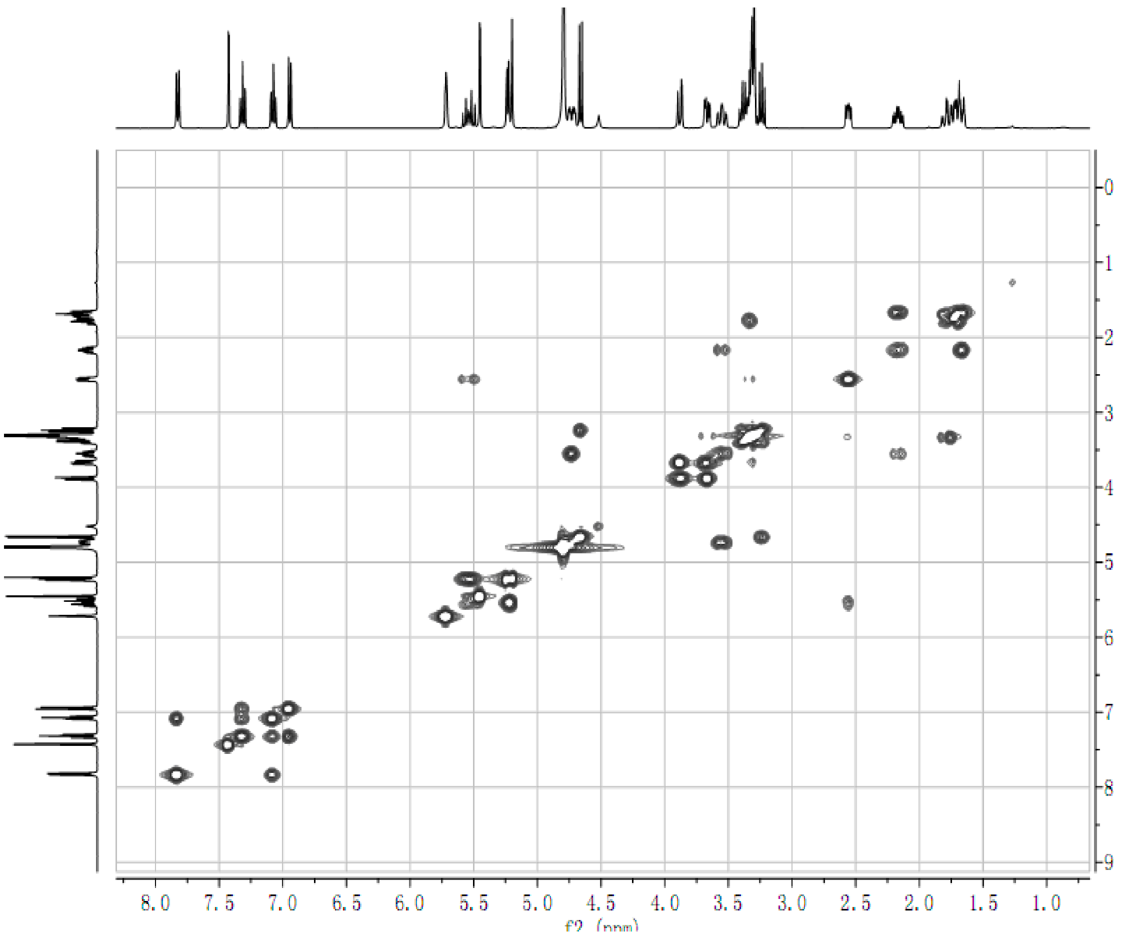

Figure S24. ^{1}H-^{1}H COSY spectrum of **6** in MeOD.

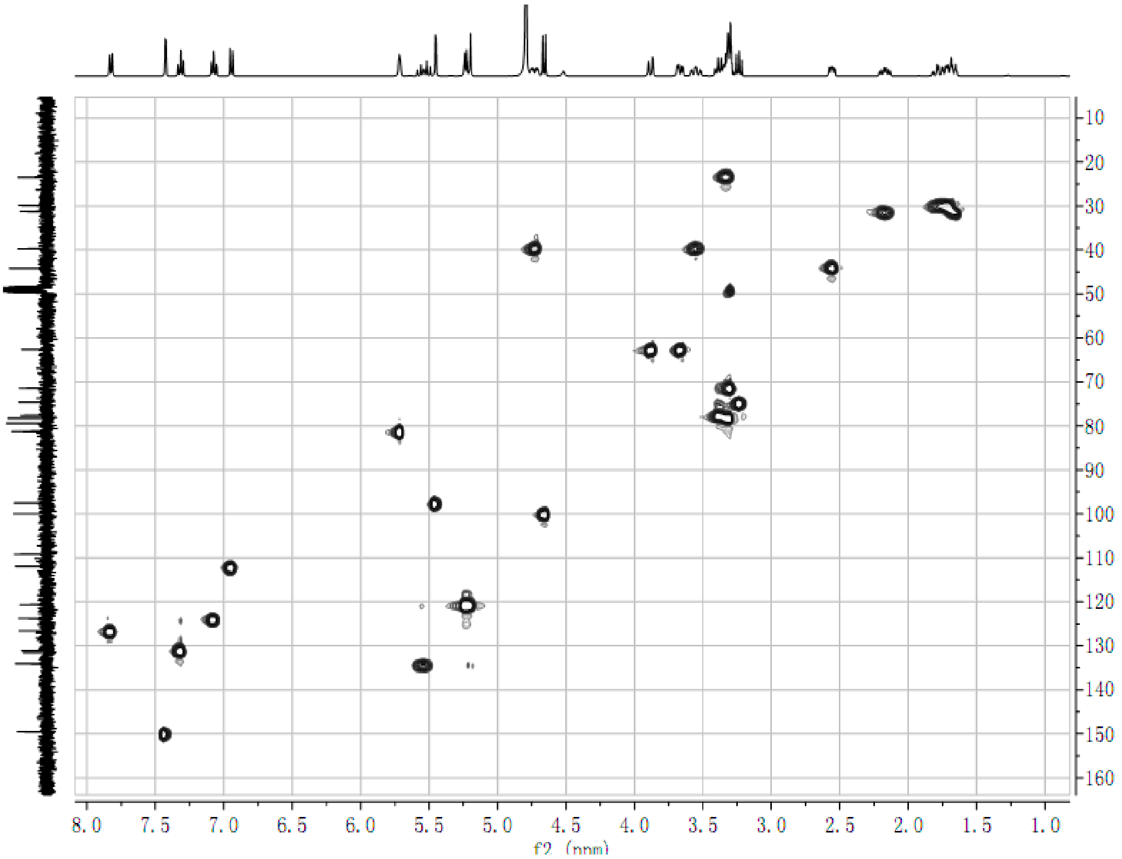

Figure S25. HSQC spectrum of **6** in MeOD.

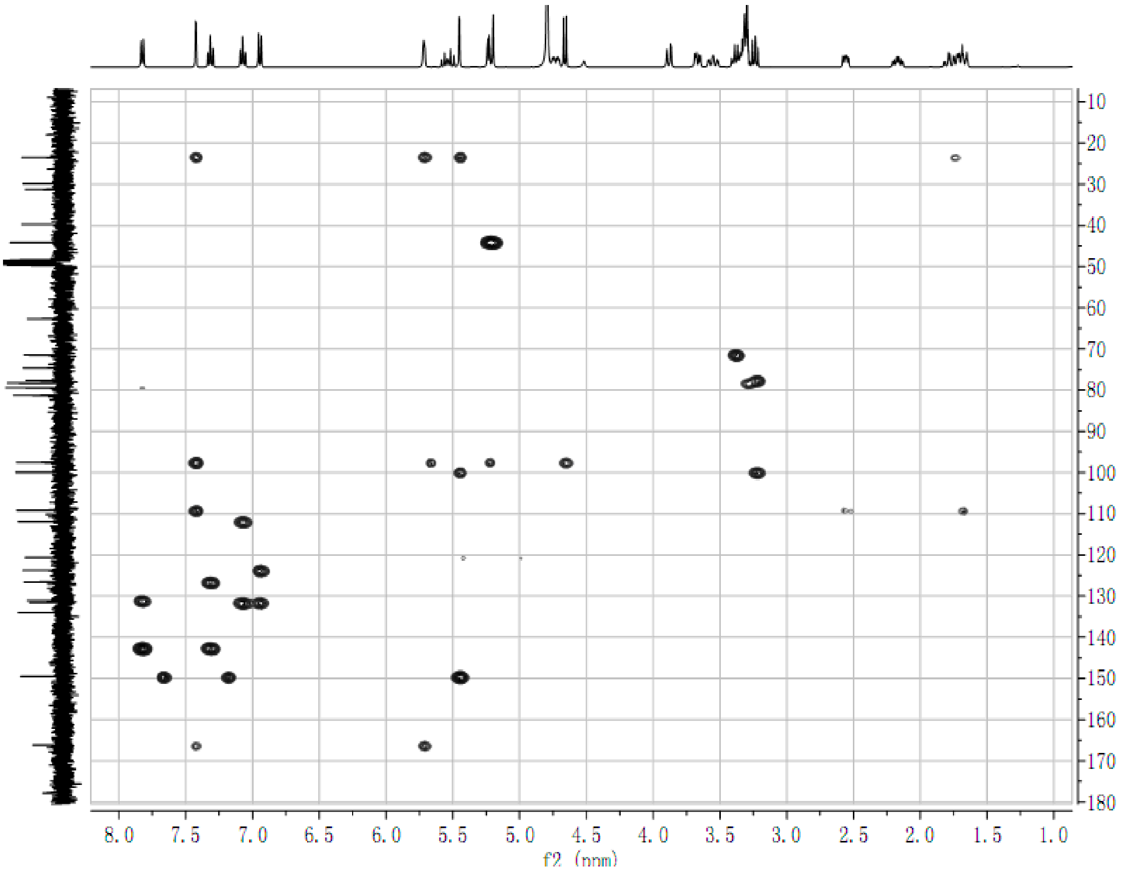

Figure S26. HMBC spectrum of **6** in MeOD.

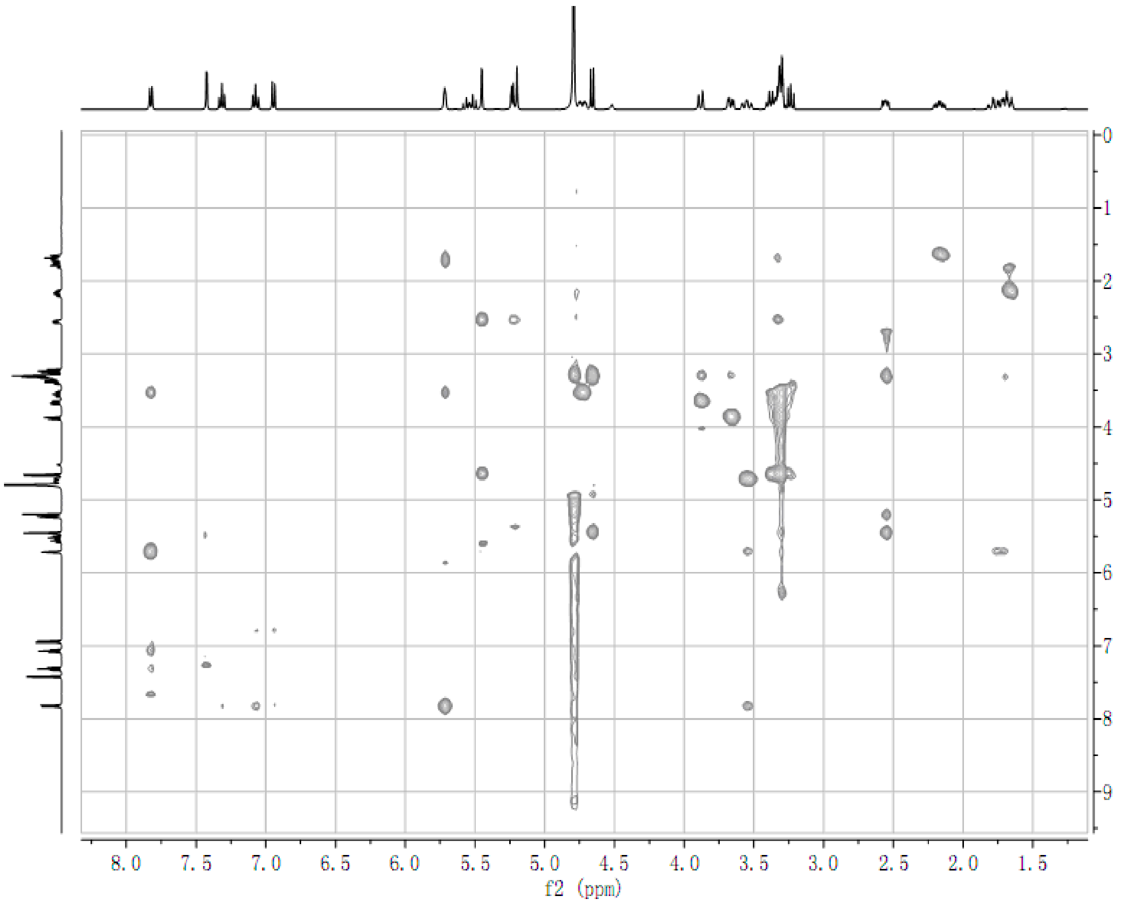

Figure S27. ROESY spectrum of **6** in MeOD.
